# Bringing the Flipped Classroom to Day 1: A Novel Didactic Curriculum for Emergency Medicine Intern Orientation

**DOI:** 10.5811/westjem.2017.11.35286

**Published:** 2017-12-18

**Authors:** Michael G. Barrie, Christopher Amick, Jennifer Mitzman, David P. Way, Andrew M. King

**Affiliations:** The Ohio State University, Wexner Medical Center, Department of Emergency Medicine, Columbus, Ohio

## Abstract

Most emergency medicine (EM) residency programs provide an orientation program for their incoming interns, with the lecture being the most common education activity during this period. Our orientation program is designed to bridge the gap between undergraduate and graduate medical education by ensuring that all learners demonstrate competency on Level 1 Milestones, including medical knowledge (MK). To teach interns core medical knowledge in EM, we reformulated orientation using the flipped-classroom model by replacing lectures with small group, case-based discussions. Interns demonstrated improvement in medical knowledge through higher scores on a posttest. Evaluation survey results were also favorable for the flipped-classroom teaching format.

## BACKGROUND

Almost all emergency medicine (EM) residencies provide orientation programs for their incoming interns.^1^ Orientation programs are commonly a mix of clinical time, didactic teaching, administrative onboarding, and social activities.^1^ While medical educators have sought to replace lectures with alternative methods that promote active learning and longer term retention,^2^–^3^ lectures continue to be the predominant educational activity during EM orientations.^1^,^4^–^5^ Few programs have yet to engage in baseline, or programmatic, assessment of Level 1 Milestones for incoming interns.^1^,^6^

More recently, flipped-classroom methods have been adopted by EM residency programs.^7^–^8^ The flipped- classroom method generally involves preparation by the learner, in the form of self-directed learning, in advance of a face-to-face classroom meeting. Class time is reserved for application of the learner’s new knowledge through facilitated discussion of problems or cases.

## OBJECTIVES

The purpose of our redesigned intern orientation program was to provide and assess intern’s core medical knowledge (Level 1 Milestone-MK) to enable them to succeed in residency. In an attempt to improve the didactic component of intern orientation, we developed case-based, pre-reading assignments organized by common EM topics. Our approach mirrored the flipped-classroom model we employ throughout our entire residency program.

## CURRICULAR DESIGN

The orientation program was six weeks in length, running from mid-June to the end of July. Core EM knowledge teaching was allocated to 21 hours of direct instruction (seven hours per week) during the last half of the program. Each hour of direct instruction time represented a topic defined by a “chief complaint.” Hour- long lectures that had previously covered this core content were replaced by case-based, interactive small group sessions.

Small group sessions were designed by core faculty and senior EM residents, and reviewed by the orientation director. Residents were expected to prepare for each session by reading a patient case and covering the prescribed learning material. Residents were provided with guiding questions for each patient case. These included questions about differential diagnoses, management, and dispositions. Residents were also encouraged to find their own resources to answer guiding questions. During the sessions, faculty members facilitated the discussion by navigating a facilitator guide that included the guiding questions. Examples of topics covered were chest pain, abdominal pain, shortness of breath, airway management, headache, and back pain, among others.

To measure knowledge gains, we contracted with TrueLearn® to generate two parallel examinations, each containing 100 randomly selected items from their SmartBank for Emergency Medicine.^9^ One examination was administered in the first week of orientation, while the other was administered during the last week. The examinations were timed (power tests) and completed online. We also implemented a program evaluation survey that included a retrospective pretest (RPT) question about resident gains in proficiency on core content covered in small group sessions.^10^ Our institutional review board declared this exempt research.

## IMPACT/EFFECTIVENESS

Twelve of 16 residents completed both pre- and posttests. Knowledge test scores were reported by TrueLearn® as percentage correct. We analyzed these with a paired t-test and Cohen’s d effect size. Interns made an average gain of 12.6 percentage points between pre- and posttests. This was considered statistically significant with an extremely large effect size (t=−6.78; df=11; p≤.001; es=−2.73; see Figure).

In response to the retrospective pretest evaluation item, eight of 10 (80%) residents said that they felt more proficient with the core content covered in the small group sessions than they did before completing orientation.

Use of the flipped classroom during our orientation had the side benefit of preparing residents for the teaching methods employed throughout residency. While knowledge test results showed significant and large learning gains, we were not able to directly attribute these gains to the flipped-classroom approach. We did not assess resident preparation for, or participation in small group discussions. However most residents rated the small group sessions as beneficial.

Other evaluation results suggested the need for faculty development with facilitating small group discussions. We also learned the importance of posting learning material on an easy-to-access online learning management platform, particularly for residents who worked clinically at dispersed sites.

We observed some preliminary evidence that the flipped classroom model is an effective teaching method that provided learners with the ability to customize their study time. This is particularly helpful during orientation for interns who come from varied medical school backgrounds. We did not, however, make a direct comparison to a lecture-based orientation. Additional evidence is needed through controlled experiments comparing lecture to flipped-classroom methods.

## Figures and Tables

**Figure:**
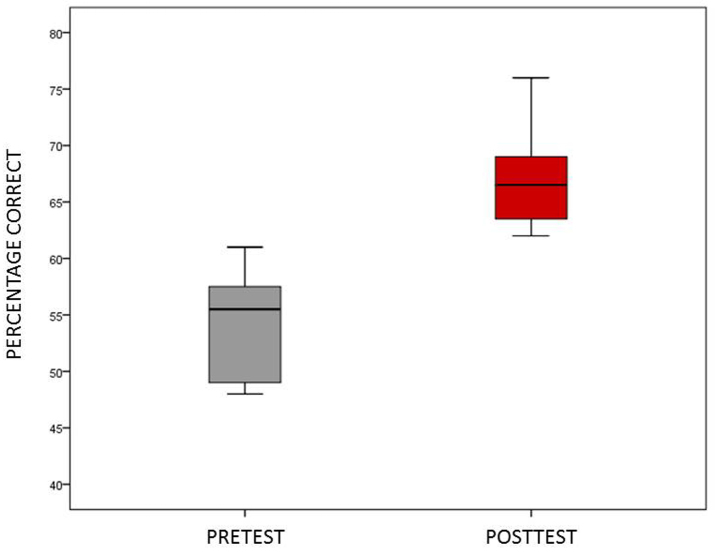
Box and whisker plot representing the median and distribution of 12 residents percentage scores on pre- and post knowledge tests generated from the TrueLearn® Smartbank.
